# Application of the Lamb Wave Mode of Acoustic Emission for Monitoring Impact Damage in Plate Structures

**DOI:** 10.3390/s23208611

**Published:** 2023-10-20

**Authors:** Baochun Xu, Jiacai Huang, Yachun Jie

**Affiliations:** Nanjing Institute of Technology, Nanjing 211167, China; y00450220301@njit.edu.cn

**Keywords:** health monitoring of plate structures, impact, damage, acoustic emission, Lamb wave

## Abstract

The impact acoustic emission (AE) of plate structures is a transient stress wave generated by local materials under impact force that contains the state information of the impacted area. If the impact causes damage, the AE from material damage will be superimposed on the impact AE. Therefore, this paper details the direct extraction of damage-induced AEs from impact AEs for the health monitoring of plate structures. The damage-induced AE was analysed based on various aspects, including the cut-off range and propagation speed characteristics of the Lamb wave mode, the correlation between the force direction and the Lamb wave mode, and the impact damage process. According to these features, the damage-induced AE wave packets were extracted and verified via impact tests on epoxy glass fibreboards. The results demonstrated the feasibility of the proposed method for determining whether an impact causes damage via the direct extraction of the damage-induced AE from the impact AE.

## 1. Introduction

Plate structures are widely used for sealing high-speed transportation systems such as aircraft, satellites, and high-speed rails. They might become damaged during service due to working loads and the impact of foreign objects, and even minor damage can lead to catastrophic events under harsh working conditions. Thus, health-monitoring systems for plate structures have been developed to forestall such events. Among them, acoustic emission (AE) technology is widely used because AEs are very sensitive in detecting damage-coupled material fracturing. Further, the non-stationary nature of AEs is associated with damage types, and AEs carry rich damage information [1,2,3]. Therefore, AE-based monitoring is extensively applied in composite performance testing and structural health monitoring. Romhány et al. [4] reviewed relevant papers from 1991 to 2017 on the use of AEs for characterising polymer composites; they found that acoustic source localisation and failure assessment of structural materials based on AEs were considered an ideal method for monitoring the health of composite structures rather than a mere testing approach for such materials. Fotouhi et al. [5] pointed out that the online monitoring of crack propagation based on AEs is an ideal approach for acquiring accurate fracture mechanical parameters of materials. Saeedifar and Zaroucha [6] reviewed the relevant literature on the damage characterisation of laminated composite materials (spanning from 1975 to 2020); they concluded that using AE technology in damage characterisation is advantageous due to its sensitivity to changes in the health states of structural materials. The above-mentioned studies show that AEs carry information on material performance and structural health. Hence, health information can be better obtained through structural health monitoring based on AEs.

In the cited works, methods such as eliminating or lowering the interference of load changes on damage-induced AEs with approximately static or low-frequency alternating loads, or reducing the interference between different damage mechanisms by setting specific experimental conditions to only produce or focus on certain damage, were adopted. Using these approaches, reliable damage-induced AE information can be obtained to study the mapping relationship between damage mechanisms and characteristics. These are all suitable experimental designs. Some damage processes, however, are beyond control in real environments, such as damage inflicted on composite plate structures under the impact of foreign objects during service. The previous studies on the impact damage monitoring of composite plate structures mostly focused on positioning technologies and impact flaw detection. Impact stress waves (i.e., AEs) are the optimal choice for determining an impact’s location. In plate structures, an acoustic source is identified by a monitoring system based on the propagation speed and time of arrival (TOA) of the AE [7,8,9,10]. The health of the impacted part can be diagnosed using other methods since AEs are applied in impact positioning in most cases. Petrucci and Dhakal [11,12] applied a quasi-static-load bending force to a polymer composite plate structure behind the impact plate structure, causing stress concentration at the damage location that might have led to an increase in damage and damage-induced AEs. The type of impact damage in plate structures can be determined by analysing their characteristics. In addition, the proposed methods are suitable for studying the material properties of plate structures and the corresponding damage development under loads after an impact. In studies on the post-impact damage of composite plate structures, researchers such as Ying and Dziendzikowski [13,14] diagnosed the impact damage in plate structures by generating $A_{0}$-mode Lamb waves in plates with actuators and collecting the scattered waves caused by the damage. Researchers such as Nardi and Frieden [15,16,17] showed that the degree and type of damage in plate structures can be identified according to the change in their vibration frequency characteristics before and after the impact damage is in inflicted. The post-impact monitoring method can undoubtedly avoid the interference of impact stress waves on defect detection, allowing one to effectively inspect invisible damage in composite plates; however, this method must be performed under specific conditions, such as with a continuous and stable tensile force, under a bending force, or in an offline status, with these option preventing online monitoring. The best strategy for the online health monitoring of plate structures is to evaluate impact damage by using impact AEs. Okafor et al. [18] conducted relevant research in 2001, reporting that the increase in AE energy alongside kinetic energy without damage was significantly higher than these increments accompanying the damage that occurred through connecting the signal energy generated by the impact with the kinetic energy of the impact. Bruno et al. [19] calculated damage parameter (DPs) using the wave packet characteristic parameter of the first arrival sensor for evaluating the delamination area resulting from a high-speed impact in research concerning the evaluation of the high-speed impact damage of composite plate structures. Saeedifar et al. [20] found that the signal characteristics of the high-frequency band (the filtering frequency band was 100–900 kHz) of low-speed-impact AEs are basically consistent with the AE characteristics of quasi-static loads, proving that they can indicate the impact damage type in the case of low-speed impacts in composite plate structures. These studies demonstrate that impact AEs carry damage information. In studies by Okafor, Bruno et al., and Saeedifar et al. [18,19,20], damage evaluation was completely dependent on the impact AE characteristics; in the first two cases, the research was conducted as per the energy characteristics of the AEs generated via kinetic energy, while such an evaluation focused on the characteristics of the impact-generated AE in the last case. Moreover, the impact plate structures of external objects are uncontrollable in engineering applications. The characteristic change in AEs depends on factors such as impact location, impact energy, and propagation distance; thus, factors unrelated to damage might affect damage monitoring. This paper suggests that eliminating the interference of AE characteristic changes caused by non-damage factors in damage monitoring is worth studying.

The influence of non-damage factors can be minimised if damage-induced AEs can be directly extracted for the health monitoring of plate structures. Neither the systematic analysis of the difference between damage and external impact AEs nor a sound basis and technical route for extracting damage-induced AEs [21,22] are available in the literature, although some studies have pointed out that the $S_{0}$ mode of damage activation can be extracted for locating damage via empirical mode decomposition. The separate extraction of damage-induced AEs in accordance with the above-mentioned works on the characteristics of high- and low-speed impacts is difficult. There are various factors to consider. An impact is a dynamic process beyond control, which might lead to diversified damage [11,12,13,14,15,16,17]. When damage-induced AEs have low energy and the AE energy generated by the impact behaviour is dominant, the abnormality of damage related to health cannot be derived from the amplitude when these two energies are superimposed [7,17,18,19]. Regarding the generation mechanisms of AEs, there is no difference between the impact of external objects and damage-induced AEs since they are both stress waves with the same frequency domain characteristics, without discrepancies in the form of propagation in the plate [4,6]. In this case, judging whether a structure is damaged based on changes in characteristics such as frequency, amplitude, and waveform does not yield convincing evidence [18,19,20]. The above-mentioned AE-based impact-damage-monitoring methods of plate structures are based on the characteristics of damage-induced AEs under a static load, but they cannot provide a basis for health diagnosis through the theoretical analysis of the characteristics of plate structures with the help of signal information extraction technologies.

In this paper, the feasibility of extracting damage information from impact AEs is demonstrated through the theoretical analysis of the AE characteristics in a plate, and this also forms the basis for selecting an appropriate signal-information-processing technology. Establishing a theory that can describe the characteristics of plate stress waves (AE) with universal significance and considers the influence of plate structure parameters on these waves is unrealistic. Nonetheless, the theoretical analysis of the stress wave characteristics in a plate for a certain plate structure is feasible. Therefore, this manuscript discusses the relationship between the force direction and the characteristics of wave velocity for the Lamb wave mode as a form of stress wave propagation in thin plate structures, which are used commonly in engineering, to interpret the AE waveform characteristics in the plate according to the instantaneous energy release of AEs. An appropriate signal-processing method is also proposed to extract the damage-induced AEs from the impact AEs as per the above characteristics for the health monitoring of plate structures.

## 2. Analysis of the Relationship between the Lamb Wave Mode and Force Direction of AEs in Plate Structures

An AE is a stress wave that propagates as a Lamb wave in thin plate structures. The relationship between the stress wave mode and force direction in a thin plate was analysed according to the Lamb wave dispersion curve; the conclusions were derived from the mechanical equations of plate structures and then verified via a lead-breaking test.

### 2.1. Analysis of the Lamb Wave Mode Characteristics in Plates

**Figure 1:**
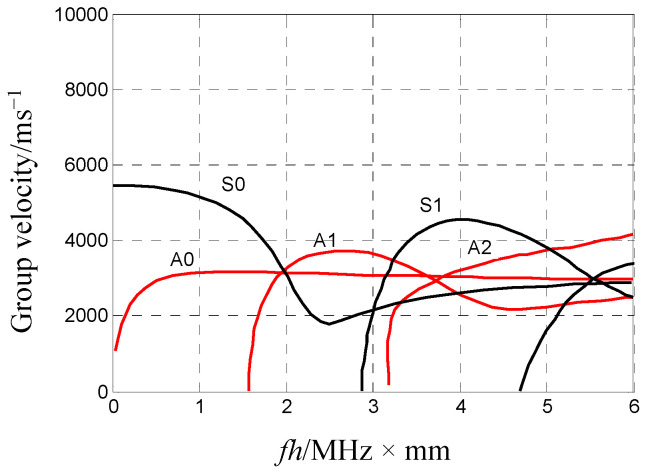
Relationship between group velocity and frequency–thickness product of Lamb waves in aluminium plates.

Lamb waves are stress waves propagating in a structure with two free parallel planes, and their wave characteristics are expressed by the Rayleigh–Lamb equation, which determines whether the Lamb wave mode is multi-mode or dispersion. There are at least two Lamb wave modes, with their propagation speeds related to the frequency at any activation frequency. A Lamb wave is divided into a symmetric (S) wave and an anti-symmetric (A) wave based on the phase relationship of the mass points on the surface of an object. Compared with isotropic material plates, composite material plates present complex wave characteristics that depend on the corresponding fibre material, fibre laying direction, and resin material. However, the main characteristics of Lamb waves are basically consistent. The relationship between the group velocity and frequency–thickness product (fh, in MHz·mm) of Lamb waves in an aluminium plate structure [21] is shown in Figure 1 to clarify their characteristics in the plate; the multi-mode and dispersion characteristics for these Lamb waves carry rich information with a high application value for plate structure health monitoring. A well-characterised Lamb wave can be selected for plate structure health monitoring; this is conducive to determining its mode and wave speed and better serves a given application.

Furthermore, if the frequency remains unchanged, the plate will become thinner as fh decreases. In this case, the labels $S_{0}$ and $A_{0}$ in Figure 1 indicate that the Lamb wave mode has been cut short. The propagation speed of $S_{0}$ and $A_{0}$ varies significantly in this mode’s cut-off range. The speed change of the $S_{0}$ mode is not highly correlated with the change in fh when fh<1, and the slight variation in the whole range can be approximated as a constant value. In contrast, the speed change of the $A_{0}$ mode is highly correlated with the change in fh and increases along with fh, showing a significant frequency dispersion.

Moreover, the characteristics of the Lamb wave mode in this mode’s cut-off range can also be found in plate structures consisting of other materials, such as composites. The $S_{0}$ and $A_{0}$ modes of a Lamb wave can be observed in a plate structure when the fh of the Lamb wave is in the modes’ cut-off ranges. Under this condition, the $A_{0}$ mode waveform should have a high frequency in the front and a low frequency behind it, indicating dispersion if the excitation source is a broadband signal. In comparison, the propagation speed of the $S_{0}$ mode tends to be constant, and its waveform should maintain the initial propagation of the generation source. The $S_{0}$ mode propagation speed is almost independent of fh in this mode’s cut-off range; therefore, some studies recommend its use for locating the acoustic source [4,22,23].

### 2.2. Analysis of the Relationship between Force Direction and Wave Velocity Characteristics

**Figure 2:**
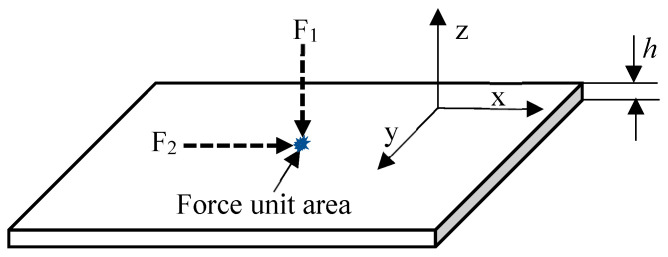
Force-activating waves in a plate structure.

An AE in a plate is an elastic wave whose vibration corresponds to classical mechanical theory. Hence, its characteristics can be analysed based on mechanical equations. The characteristics of waves generated by forces perpendicular and parallel to a plate are defined by the balance equation of force and the motion equation of particles [24,25]. Assuming that the plate has a thickness of h, the force being applied to its structure unit is F_1_ in the vertical direction and F_2_ in the horizontal direction (Figure 2).

If the force direction is perpendicular to the plate’s surface, the balance equation of the plate can be simplified as

(1) $$D\Delta^{2}\zeta -P=0$$

where D is the bending strength of the plate, P is the force acting on the unit area of the plate surface, ζ is the displacement per unit area under the force, and Δ is the Laplacian Operator, which is $\Delta =d^{2}/dx^{2}$ under one-dimensional conditions. According to the definition of bending strength, the following equations can be obtained from the relationship between force and displacement:

(2) $$D=Eh^{3}/12(1-\sigma^{2})$$

and

(3) $$P=-\rho h\partial^{2}\zeta /\partial t^{2}$$

Here, E is Young’s modulus, σ is the Poisson’s ratio, ρ is the plate density, and ρh is the mass per unit area. By substituting Equations (2) and (3) into Equation (1), the free vibration of the plate can be expressed as

(4) $$\rho h\partial^{2}\zeta /\partial t^{2}+Eh^{3}/12(1-\sigma^{2}){∆}^{2}\zeta =0$$

The solution to Equation (4) is discussed within the harmonic range. Hence, ζ can be written as $\zeta_{0}exp\left[j(kx-\omega t)\right]$, where $\zeta_{0}$ is constant. By discussing a one-dimensional problem, $\Delta^{2}\zeta$ and $\partial^{2}\zeta /\partial t^{2}$ in Equation (4) can be written as

(5) $$\Delta^{2}\zeta =\partial^{4}\zeta /\partial t^{4}=k^{4}\zeta_{0}exp\left[j(kx-\omega t)\right]$$

and

(6) $$\partial^{2}\zeta /\partial t^{2}={-\omega }^{2}\zeta_{0}exp\left[j(kx-\omega t)\right]$$

By substituting Equations (5) and (6) into Equation (4), the last can be simplified as

(7) $$\rho (-\omega^{2})+Eh^{2}k^{4}/12(1-\sigma^{2})=0$$

(8) $$\omega =hk^{2}{\left[E/12\rho (1-\sigma^{2})\right]}^{1/2}$$

or

(9) $$k={\left[12\rho \omega^{2}(1-\sigma^{2})/Eh^{2}\right]}^{1/4}$$

The wave phase velocity (c=ω/k) can be derived from Equation (9) as follows:

(10) $$c={\left[{Eh}^{2}/12\rho (1-\sigma^{2})\right]}^{1/4}\sqrt{\omega }={\left[E/12\rho (1-\sigma^{2})\right]}^{1/4}\sqrt{\omega h}$$

The wave group velocity ($c_{g}=\partial \omega /\partial k$) can be obtained from Equations (8) and (9) as follows:

(11) $$\begin{array}{c} c_{g}=2hk{\left(E/12\rho (1-\sigma^{2})\right)}^{1/2}=2h{\left[12\rho \omega^{2}(1-\sigma^{2})/Eh^{2}\right]}^{1/4}{\left[E/12\rho (1-\sigma^{2})\right]}^{1/2}\\ ={\left[4E/3\rho (1-\sigma^{2})\right]}^{1/4}\sqrt{\omega h} \end{array}$$

The generated wave propagates along the axis direction if the force direction is parallel to the plate surface. However, the mass–point displacement propagates in both the y-axis and x-axis directions. Thus, the motion equations of the two directions can be presented as

(12) $$\left(\rho /E\right)\partial^{2}u_{x}/\partial t^{2}=\left[1/\left(1-\sigma^{2}\right)\right]\partial^{2}u_{x}/\partial x^{2}+\left[1/2\left(1+\sigma \right)\right]\partial^{2}u_{x}/\partial y^{2}+\left[1/2(1-\sigma )\right]\partial^{2}u_{y}/\partial x\partial y$$

and

(13) $$\left(\rho /E\right)\partial^{2}u_{y}/\partial t^{2}=\left[1/\left(1-\sigma^{2}\right)\right]\partial^{2}u_{y}/\partial y^{2}+\left[1/2\left(1+\sigma \right)\right]\partial^{2}u_{y}/\partial x^{2}+\left[1/2(1-\sigma )\right]\partial^{2}u_{x}/\partial x\partial y$$

If the wave propagating along the x-axis is considered alone while neglecting coupling, the following can be obtained:

(14) $$\partial^{2}u_{x}/\partial t^{2}=\left[E/\rho \left(1-\sigma^{2}\right)\right]\partial^{2}u_{x}/\partial x^{2}$$

and

(15) $$\partial^{2}u_{y}/\partial t^{2}=\left[E/2\rho \left(1+\sigma \right)\right]\partial^{2}u_{y}/\partial x^{2}$$

Then, the wave velocity of particle displacement along the x-axis is expressed as

(16) $$c_{ext}={\left(E/\rho \left(1-\sigma^{2}\right)\right)}^{1/2}$$

while that along the y-axis is given by

(17) $$c_{t}={\left(E/2\rho \left(1+\sigma \right)\right)}^{1/2}$$

The propagation speed of the wave generated by the force in the plate is related to the force direction, and according to the results obtained from the above equations, there are, theoretically, three waves with different velocity characteristics. The propagation speed of the wave generated by the force perpendicular to the plate direction is shown in Equation (11), and the corresponding wave velocity is associated with the frequency. The propagation speed of the wave generated by the force parallel to the plate is shown in Equations (16) and (17), and the wave velocity is independent of the frequency. Note worthily, the characteristics of the plate structure material discussed above are isotropic or approximately isotropic. If the characteristics of the plate structure material are anisotropic, the expression of the wave speed will differ, but the relationship between the propagation speed and frequency of the wave excited by the force in different directions is still valid [26,27].

### 2.3. Analysis of the Relationship between Force Direction and AE Mode

An AE is a transient elastic wave generated by the dynamic change of local areas after a structure is subjected to external conditions, such as the impact of external objects and alternating loads. Therefore, AEs carry health status information about a structure pertaining to the acoustic source. However, an AE is a transient elastic wave with an uncontrollable behaviour and a wide frequency band, and its spreading within a plate is in the form of multi-mode or dispersion Lamb waves. In other words, the information carried by an AE about the health status of a plate structure cannot be interpreted easily due to its complex waveform. Hence, this study’s authors considered that basic support from theoretical analysis is essential for interpreting such information in a specific and reliable manner. However, the adoption of a universally applicable theory is not realistic since the characteristics of AE propagation in a plate are related to its structural parameters, for which there are complex types. The Lamb wave has a mode cut-off range with only two basic modes, $S_{0}$ and $A_{0}$, according to the Lamb wave mode characteristics analysed above. If the Lamb waveform is in this range, the corresponding AE information is simple and not influenced by other modes. Thin plate structures have a certain application value [7,13]. Thus, in this study, a plate structure with a thickness not greater than 2 mm was considered. Since the AE signals in plate structures are broadband, i.e., mainly below 500 kHz [6], the product of frequency and plate thickness in this study is less than 1 MHz∙mm, which is within the mode cut-off range. That is, an AE waveform and its carried information can be interpreted based on the propagation characteristics of the $S_{0}$ and $A_{0}$ modes.

**Figure 3:**
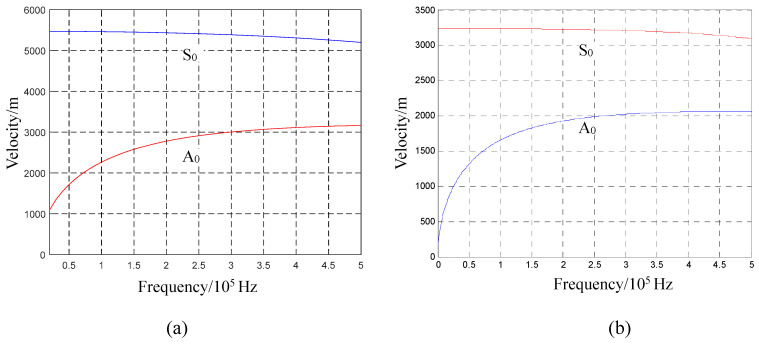
Zero-order-mode group velocity curve of the Lamb wave in 2 mm thick plate structures when the frequency is below 500 kHz (**a**) in an aluminium plate and (**b**) in epoxy–polyester fibreglass.

According to the definition of an AE, an acoustic source in a plate structure originates from the action of a force, which might result from external events, such as the impact of external objects, or internal events, such as plate fracturing. The different characteristics of wave velocity generated by vertical and parallel forces are proven by Equations (11), (16) and (17). The AEs generated in the plate structure under the action of a force should also conform to this characteristic, and it propagates as a Lamb wave in the plate. Further characteristic analysis shows that the relationship between the force direction and Lamb wave mode can be established for the plate structure. Equation (17) refers to a shear horizontal (SH) wave according to particle vibration form and wave velocity, but an SH wave cannot be obtained from the conversion of the piezoelectric effect because its energy is low in the signal collected by a piezoelectric device [28,29]. Moreover, such an SH wave neither overlaps with the Lamb wave mode nor participates in its conversion [25]. Therefore, the SH wave is ignored in an AE, conforming to the methods adopted in previous works [4,26,30,31]. Hence, the corresponding relationship that must be discussed is only that between the stress wave (see Equations (11) and (16)) and the Lamb wave mode. The fh in a thin plate structure is within the mode cut-off range of the Lamb wave, as explained above. Equations (11) and (16) regarding force activation in the thin plate should correspond to the $S_{0}$ and $A_{0}$ modes since, based on the interpretation of the Lamb wave mode, these are the only AE modes in the thin plate. In Equation (11), the velocity of the bending wave is related to the frequency. Equation (16) refers to an extended wave that is a symmetric mode of the Lamb wave, which is independent of frequency according to the particle displacement in Equation (11). Figure 3 displays the $S_{0}$ (when fh < 1 MHz·mm) and $A_{0}$ modes of a 2 mm thick aluminium plate and epoxy–polyester fibreglass in the mode cut-off range. The wave velocity of the $A_{0}$ mode monotonically increases along with the frequency, exhibiting frequency dispersion; the wave velocity of the symmetric wave mode, in contrast, is almost constant. Based on this, Equation (11) agrees with the $A_{0}$ mode characteristics of Lamb waves, while Equation (16) is consistent with the characteristics of their $S_{0}$ mode.

Therefore, the AEs generated by the force that is perpendicular to the plate surface and those generated by the force parallel to the plate correspond to the $A_{0}$ and $S_{0}$ modes, respectively.

### 2.4. Mode Verification of AE Force Excitation in the Plate Structure

The force direction in the plate structure determining the Lamb wave mode of an AE can be explained theoretically based on the analysis provided above. This was verified by studying a 2 mm thick aluminium plate and a composite plate. The force-generated AE in the plate structure was stimulated through the common lead-breaking test as follows [4,31]. A piece of lead was broken by pressing its core on the end face and surface of the plate to simulate AE activation via forces parallel and perpendicular to the plate surface, respectively. The sensor adopted was a piezoelectric ceramic sensor (PZT) with a diameter and thickness of 8 mm and 0.5 mm, respectively. It was pasted on the plate structure surface and had a signal acquisition frequency of 10^7^ Hz. Various PZTs were placed on the propagation path, in line with the lead breaking position, to observe the change in the AE waveform of each lead piece breaking in the propagation process. The lead-breaking position on the upper surface of the plate structure was close to the end face to ensure that this distance was equal to the distance from the lead-breaking positions to the sensor surface.

**Figure 4:**
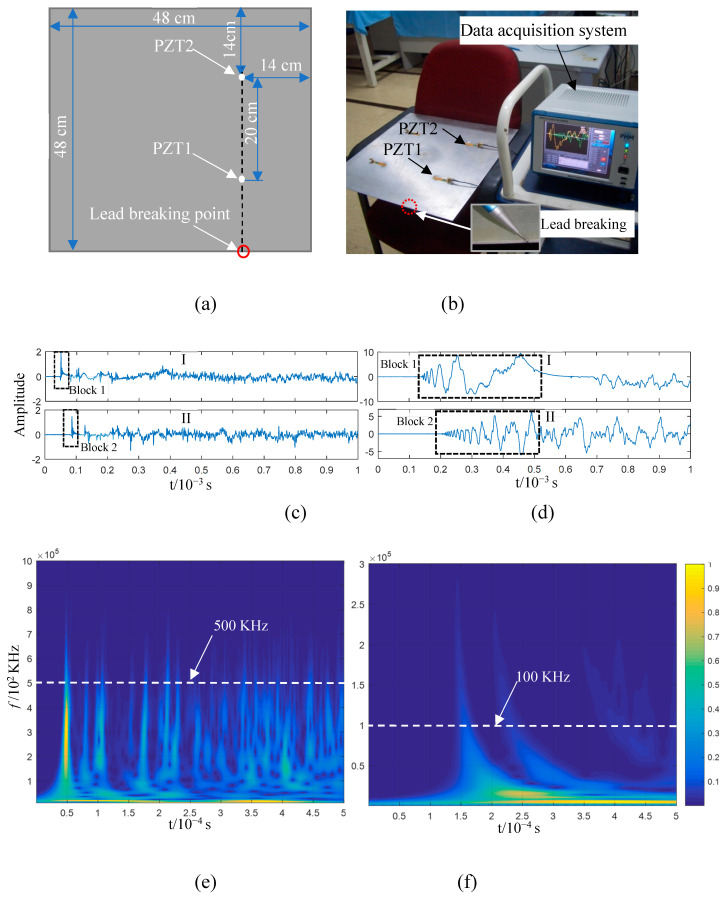
Lead-breaking test conducted on an aluminium plate structure: (**a**) experimental layout and (**b**) setting; (**c**,**d**) signals collected from the piezoelectric ceramic sensors PZT1 and PZT2 at the end face (**c**) and upper surface (**d**); (**e**,**f**) wavelet time–frequency analysis of the first 5000 points of the PZT1 signal, with lead breaking at the end face (**e**) and upper surface (**f**).

Figure 4a,b illustrate the lead-breaking test conducted on an aluminium plate, which was 48 cm in length and width, including the positioning of the PZTs on the aluminium plate; PZT1 and PZT2 were 20 cm apart and 14 cm away from the plate edge. Figure 4c,d display the AE signals generated via breaking lead, with 10^4^ acquisition points. The signal acquired from lead breaking at the end face of the plate structure consisted of sub-graphs I and II, which corresponded to the AE signals collected from PZT1 and PZT2, respectively. In sub-graph I, the AE impact wave reaching PZT1 is indicated by Block 1; it remained in the impact state while reaching PZT2 after a 20 cm propagation, as shown by Block 2 in sub-graph II. Considering wave reflection and attenuation, the wave packet that reached the sensor first was the least likely to be contaminated, with the frequency component being the closest to the initial state. Hence, the time–frequency information of the first 5000 points in sub-graph I of Figure 4c was analysed using a wavelet transform, as shown in Figure 4e. The main frequency components were distributed in the range below 500 kHz when an AE was generated via lead breaking at the end face (Block 1 in Figure 4c). The impact wave in Box 1 was composed of abundant frequency components, which kept the wave in a state similar to that detected by PZT1 after propagation within a certain distance and the arrival of PZT2, although there are abundant frequency components of the impact wave in Block 1. This shows that the propagation speed of waves with different frequencies is consistent in an AE, and that is why the initial impact waveform can be maintained in the propagation. If the maximum points of the wave packet indicated by Block 1 and Block 2 in Figure 4c are taken as the reference points, the time difference between the two impact wave packets is approximately 3.69 × 10^−5^ s. Based on this, the wave velocity can be calculated as 5420 m/s, and the distance between the two PZTs is 20 cm. This is close to the $S_{0}$ mode wave velocity described in Figure 3a. The propagation characteristics and speed of wave propagation indicated by Blocks 1 and 2 in Figure 4c indicate that the $S_{0}$ mode is the AE generated via lead breaking at the end face of the aluminium plate.

Figure 4d shows the AE signal obtained when the lead was broken on the upper surface of the plate structure near the end face. The AE generated via lead breaking, which is a transient event, is transient and a part of the broadband spectrum, which can be verified via the lead breaking experiment at the end face (Figure 4c,e). The waveform generated through lead breaking on the upper surface of the aluminium plate first emits a high frequency and then a low frequency after reaching PZT1 after propagating for some distance, according to sub-graph I in Figure 4d, which is more evident after reaching PZT2 via 20 cm propagation, as shown in sub-graph II. The waveform characteristics in Figure 4d show that the propagation speed of the AE generated through lead breaking on the upper surface is characterised by frequency dispersion and frequency dependency. The first 5000 points of the PZT1 signal were used for a wavelet time–frequency analysis. As shown in Figure 4f, the AE frequency ranges from 0 to 100 kHz, and the time–frequency of the signal is distributed in the shape of an arc, with the high-frequency wave reaching PZT1 before the low-frequency one. This experiment proves that the AE signals collected by PZT1 and PZT2 are consistent with the characteristics of the $A_{0}$ mode shown in Figure 3a. Based on the time–frequency analysis in Figure 4f, the main frequency band of the signal is lower than 100 kHz, with the thickness of the aluminium plate being 2 mm, and the AE generated via lead breaking is in the mode cut-off range. Apparently, in this experiment, the $A_{0}$ mode corresponded to the AE generated via lead breaking on the upper surface of the aluminium plate.

**Figure 5:**
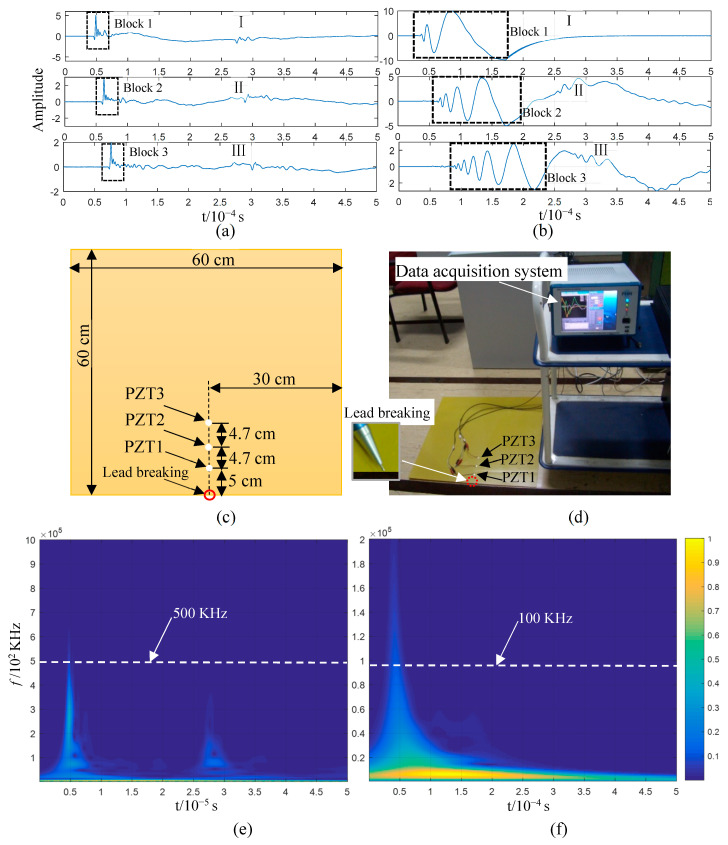
Lead-breaking test conducted on epoxy fibreglass plate structure: (**a**) experimental layout and (**b**) setting; (**c**,**d**) signal collected from the piezoelectric ceramic sensors PZT1, PZT2, and PZT3 at the end face (**c**) and on the upper surface (**d**); (**e**,**f**) wavelet time–frequency analysis of the first 5000 points of the PZT1 signal with lead breaking at the end face (**e**) and upper surface (**f**).

Figure 5 illustrates the lead-breaking test performed on the 2 mm thick epoxy fibreglass plate, whose side length was ~60 cm. Three PZTs were placed on the plate’s centre at intervals of 4.7 cm along a straight line with respect to the lead-breaking point; PZT1 was placed ~5.0 cm away from the plate end face. The waveform indicated by Block 1 in Figure 5c is distributed in a frequency band below 500 kHz; it remains almost unchanged after the wave packets marked in Blocks 1 to 3 pass through PZT1, PZT2, and PZT3. This indicates that the propagation speed of the varying frequency components of the waveform was consistent. If the maximum points of the wave packets in Figure 5c are taken as the reference points, the propagation time from PZT1 to PZT3 is ~2.72 × 10^−5^ s, with a distance of 9.4 cm. Based on this, the wave velocity can be calculated as 3460 m/s. The characteristics and propagation speed of the wave packets in Figure 5c are perfectly consistent with the $S_{0}$ mode in Figure 3b. According to these results, the AE generated via lead breaking at the end face of the epoxy fibreglass plate corresponded to the $S_{0}$ mode.

The AE waveform generated via lead breaking at the upper surface and collected by PZT1 was not fully expanded due to the short distance (Figure 5d). It continued to spread for some distance, eventually reaching PZT2 and PZT3. Then, it fully expanded, and the high-frequency component was clearly faster than the low-frequency one. The primary frequency of the signal was distributed in the frequency range of 0–100 kHz (Figure 5f). The waveform variation in the propagation process shown in sub-graphs I–III of Figure 5d indicates that the AE was characterised by dispersion. Considering that the fh in the plate is in the cut-off range of the Lamb wave mode, it can be concluded that the $A_{0}$ mode generated an AE at the lead-breaking surface of the epoxy fibreglass plate.

The propagation characteristics of the Lamb wave velocity of the $S_{0}$ and $A_{0}$ modes can perfectly explain the AE waveform generated via lead breaking based on the above tests on two plate structures. They also confirm that the $S_{0}$ mode wave velocity of the Lamb wave in the plate structure was approximately constant under the effect of a low frequency–thickness product, while the $A_{0}$ mode wave velocity exhibited significant dispersion. The conclusion in the previous subsection is supported by these test results. That is, in thin plate structures, the AE generated by the force parallel to the plate surface is in the $S_{0}$ mode, while that generated by the force perpendicular to it is in the $A_{0}$ mode. This conclusion provides strong support for extracting damage-induced AEs from plate structure impacts.

## 3. Analysis of AE Mode Information in Plate Structures

The force acting on the plate structure is the AE source, and it can be divided into an external force (OP) and an internal force (IP). The OP refers to the impact of external matter, such as ice, stones, and birds, on the plate structure, while IP indicates material fracturing, such as matrix fractures, fibre fractures, and adhesive failures, in the plate. The OP-generated AE is dominated by the $A_{0}$ mode, while the IP-generated AE is dominated by the $S_{0}$ mode [26,30,31]. The results of this study are consistent with these findings. However, the reasons for drawing this conclusion should be discussed in detail to illustrate the rationality of the technical route adopted for research purposes. Regarding the IP, with random fracture direction and size, the generated AE should not be dominated by a certain mode. Some damage types are dominated by a certain mode, but multiple damage types coexist under the material deformation, extrusion, and fracturing resulting from the impact. In general, the damage caused by an impact has the same magnitude in the parallel and perpendicular directions simultaneously, generating the $S_{0}$ and $A_{0}$ mode waves. Nonetheless, various AE frequency components of the $S_{0}$ mode maintain the same waveform in the propagation process, with concentrated energy and abundant high-frequency components due to the instantaneous nature of AEs and the mode approaching the constant propagation speed under a low frequency–thickness product. Researchers can visually observe the symbolic waveform to facilitate the study of its characteristics, but no symbolic waveform can be determined for tracking research since there is frequency dispersion in the propagation of the $A_{0}$ mode. Specifically, frequency components are distributed on the time-axis from high to low, with the energy distributed among various frequency components and different waveforms at varied positions on the same propagation path. In this way, researchers might ignore the symbolic waveform since their attention is caught by the $S_{0}$ mode with an unchanged waveform during propagation. Therefore, the authors conclude that the AE generated by an IP is dominated by the $S_{0}$ mode. The fact that OP-generated AEs are dominated by the $A_{0}$ mode might be related to the plate’s design. The plate structure’s surface, in general, is flat and smooth. When it is impacted by external objects, the component force of the impact parallel to the plate surface is relatively small or even negligible due to the surface’s flatness and smoothness. In an AE, the wave energy (amplitude) of the $A_{0}$ mode is dominant since the plate structure is subjected to a force perpendicular to its surface, and the wave energy of the $S_{0}$ mode is smaller. In that case, only the $A_{0}$-mode waveform with high amplitude can be observed, leading to a failure to recognise the $S_{0}$-mode waveform. This is different from the AE generated by impact damage. Based on the above analysis, the author of this paper considers it reasonable to conclude that the OP- and IP-generated AEs are dominated by the $A_{0}$ and $S_{0}$ modes, respectively, after excluding the specific force actions that can induce certain types of damage under laboratory conditions.

**Figure 6:**
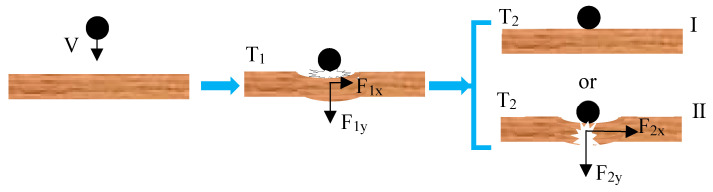
Process of a plate structure experiencing an impact.

According to the above analysis, the obtained AE signal is the superposition of the OP- and IP-generated AEs if the plate damage is caused by the impact of external objects. The amplitude of the IP-generated AE (damage) is far less than that of the OP-generated one, considering that external matter can only damage a plate structure with high kinetic energy. Moreover, there is essentially no difference between the two acoustic sources. Diagnosing whether damage is caused directly by the waveform variation of the impact—as well as determining, without sufficient analysis, that an impact AE is the cause, even if it is judged from a certain characteristic quantity of the impact AE—is difficult. This AE generated by damage might be recognised if the impact AE is interpreted based on various factors, such as the characteristics of the mode’s wave velocity, the mode’s frequency band, the relationship between force direction and mode, and the damage process induced by the impact in the mode cut-off range. When an external object impacts the plate structure, its kinetic energy is absorbed by the latter and converted into potential energy, leading to the deformation of the plate structure. This energy conversion process is realised by a force, more specifically, an external force, as shown in Figure 6. The generated internal force will cause fracturing if the deformation exceeds the bearing capacity of the plate structure, as shown in sub-graph II of Figure 6. There is a time difference between the appearance of the external and internal forces; undoubtedly, there is also a time difference for the corresponding AE signals. Note that the $S_{0}$ mode wave cannot be superimposed with the $A_{0}$ mode wave when the two modes are generated simultaneously since the wave velocity of the former is greater than that of the latter. That is, the OP-generated AE is dominated by the $A_{0}$ mode. Even if there is a low-energy $S_{0}$-mode wave, this cannot be superimposed on the $A_{0}$ mode wave. As the IP occurs later than the OP, the IP-generated AE also arises later than the OP-generated AE; however, the $S_{0}$ mode velocity is greater than the $A_{0}$ one and far greater than the low-frequency component of the $A_{0}$ mode. In this case, the AE of the $S_{0}$ mode generated alongside damage might be superimposed with that generated by OP. The AE of the $S_{0}$ mode extracted from that of the $A_{0}$ mode generated by an impact can only be generated by damage. Since damage-induced AEs continue to occur in the impact process, which is beyond control, the corresponding $S_{0}$-mode wave should also be extensively distributed in the OP-generated AE waveform.

The following conclusions can be drawn from the above considerations. First, the waveforms of the $S_{0}$ and $A_{0}$ modes generated simultaneously by OP are separated since the velocity of the former exceeds that of the latter. Therefore, the $S_{0}$-mode wave extracted from the AE of the $A_{0}$ mode generated by the OP can only be the damage-induced AE. Moreover, the $S_{0}$-mode wave has concentrated energy and several high-frequency components, whereas the $A_{0}$-mode wave features dispersion, with the energy distributed in the low-frequency range; the different frequency band characteristics of the two mode waves are conducive to their separation. Moreover, the $A_{0}$ mode of the OP-generated AE has an absolute position in terms of energy, although its AE is also induced by damage. In this case, the evidence regarding whether the $A_{0}$-mode wave is superimposed in the impact AE as the indicator of damage is not convincing. Hence, this paper proposes that the superimposition of the $S_{0}$ mode in the impact AE wave should be considered as the indicator of damage inflicted on the plate structure. Appropriate signal-processing technology must also be selected to extract indicators of damage. With its instantaneous nature, the impact AE wave is characterised by several high-frequency components. Since the $S_{0}$ mode has weak frequency dispersion under a low-thickness–frequency product, the impact waveform in the AE propagation mode is almost unchanged with concentrated high-frequency energy. Since the $A_{0}$ mode has a strong frequency dispersion, its AE energy is distributed in the low-frequency range, with little high-frequency energy. A high-frequency filter was selected here to facilitate the illustration of the $S_{0}$ mode components in the AEs. Furthermore, the time sequence information of the high-frequency wave packet extracted from the AEs was used for recognising the Lamb wave pattern and confirming the acoustic source, and its original phase information was retained. Therefore, the $S_{0}$-mode signal components of the AEs were extracted in this study using zero-phase high-pass filtering technology.

## 4. Application of Impact AE in Plate Structure Health Monitoring

Damage information can be extracted from an impact AE wave packet through multi-angle interpretation under the effect of the low-frequency–thickness product, according to the above-stated conclusions. These conclusions were successively verified by processing experimental data on plate structure damage based on the proposed technical route. Three tests were conducted: the first test aimed to verify the AE mode generated by plate structure extrusion, while the second and third ones were plate structure impact tests. The data from the last two tests were then interpreted with respect to various aspects based on the proposed technical route; this enabled the assessment of the feasibility of extracting $S_{0}$-mode information from AEs as an indicator of damage.

### 4.1. Analysis of the AE of Plate Impact Damage

**Figure 7:**
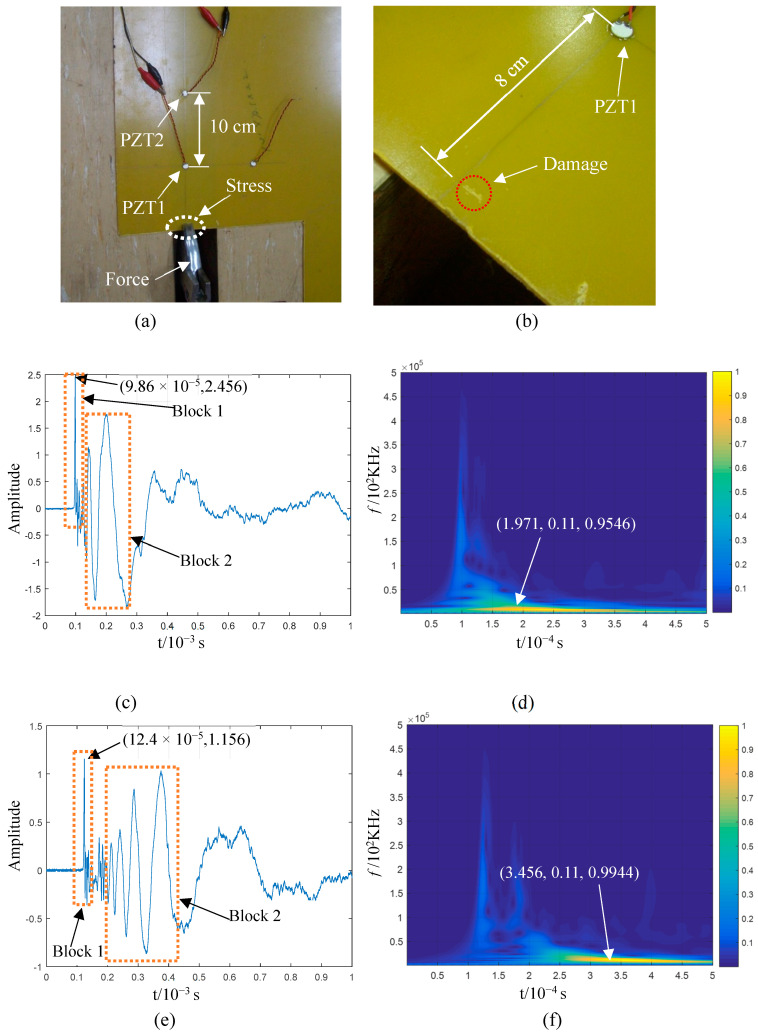
Acoustic emission (AE) signals generated via the local damage of the epoxy fibreglass plate and its wavelet time–frequency analysis: (**a**) local force of epoxy plate and piezoelectric ceramic layout; (**b**) damage area on the epoxy plate; (**c**) AE signal obtained by PZT1 and (**d**) corresponding wavelet time–frequency analysis of the first 5000 points; (**e**) AE signal obtained by PZT2 and (**f**) corresponding wavelet time–frequency analysis of the first 5000 points.

The AEs generated by impact damage inflicted on the plate structure were observed since it they are related to the feasibility of the proposed technical route. The damage-induced AE results from the fracturing of the plate structure, according to the impact damage process described above. Hence, the analysis of the AE generated by impact damage is equivalent to the direct analysis of the AE generated by material fracture. The deformation and extrusion of the plate structure, with local absorption of impact kinetic energy, were simulated in a test under the application of a local force; to eliminate the interference caused by OP, the applied force was close to the static state. The AEs that propagated in the plate resulted from its rupture, separation, and fracture, and these characteristics are similar to the damage-induced AEs caused by impacts. A 2 mm thick epoxy fibreglass plate was tested by using PZTs as sensors (Figure 7); PZT1 was placed 10 cm away from PZT2, and the force area was located along the extension line of the two PZTs.

Local damage occurred after the plate structure’s edge, which was roughly 8 cm away from PZT1, was stressed (Figure 7b). The AE signals collected by PZT1 and PZT2 are shown in Figure 7c,e; the waveform indicated by Block 1 is a shock waveform that remained almost unchanged during its propagation from PZT1 to PZT2. Then, the first 5000 points of these signals were taken for the wavelet time–frequency analysis, and the results are shown in Figure 7d,f. The frequency components of the impact waveform are distributed in the frequency range below 500 kHz. If the maximum points of the two impact wave packets are taken as the reference points, their corresponding propagation time is 25.4 μs. With a propagation distance of 10 cm, the calculated propagation speed of the wave packet is ~3900 m/s. The shock wave packet indicated by Block 1 in Figure 7c,e is a part of the $S_{0}$ mode from the perspectives of wave propagation change, fh, and wave velocity. The measured wave velocity (3900 m/s) in this mode is rather large (similar to the wave velocity in the mode cut-off range in Figure 3b) because the AE’s acoustic source was not strictly in a straight line with respect to the two sensors. Figure 7b shows that the damaged area deviating from the straight line of the two sensors resulted from the force area deviation due to uneven gripping. Moreover, the damage was inflicted over an area; thus, even if it passed through the straight line between the two sensors, the location of the acoustic source on this line could not be strictly guaranteed. Therefore, the time difference from the measured wave packet to the two sensors is less than the theoretical time difference, resulting in a higher calculated velocity. The anisotropy of the composite materials in the epoxy plate is another reason for this; although the propagation velocity of the Lamb wave in the plate can be approximately isotropic, it differs between different propagation directions.

The waves indicated by Block 2 in Figure 7c,e represent propagation dispersion, and the corresponding propagation speed decreased with the decline in frequency; this behaviour is consistent with the feature of the $A_{0}$ mode under the influence of a low-frequency–thickness product (shown in Figure 3b). The propagation velocity of the wave in Block 2 was then estimated for further verification. The local maximum of the time–frequency curve was set as the reference point by considering the points with a frequency of 11 KHz, which are the two points marked in Figure 7d,f. The time difference between the two points is ~1.485 μs, with a propagation distance of 10 cm. Further, the wave velocity is ~670 m/s. The speed approaches the wave velocity of 680 m/s, corresponding to 11 KHz (Figure 3b). The dispersion characteristics and wave velocity prove that the mode in Box 2 is $A_{0}$.

According to the above test, there exist $S_{0}$ and $A_{0}$ modes in the AEs when the 2 mm thick epoxy fibreglass plate is partially fractured by the extrusion material. Further, both modes exhibit obvious waveforms. The results show that the damage caused by the local extrusion of the plate structure has the comprehensive characteristics of multiple damage types, and the resulting force exists in two directions simultaneously; no force is absolutely dominant in a given direction. Therefore, the conclusion concerning the simultaneous existence of $S_{0}$ and $A_{0}$ modes in AEs generated through the impact damage of the thin plate structure is consistent with practical situations.

### 4.2. Extraction of Damage Information from Impact AE in Thin Plate Structures

**Figure 8:**
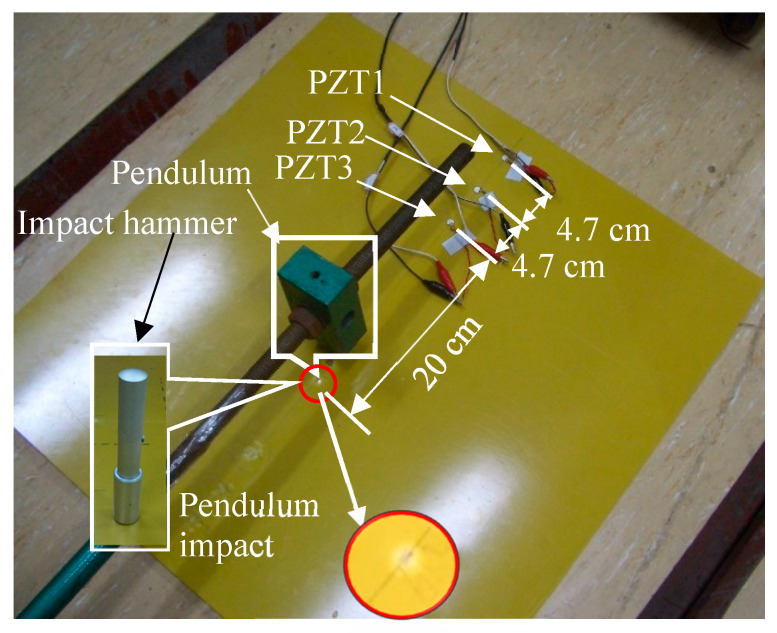
Layout of the impact damage test.

The above-mentioned results show that the impact damage of a thin plate structure is caused by a combination of AE modes. Two tests were successively conducted, one without damage in the impact and the other with impact damage. Then, by processing the test data according to the proposed technical route, the $S_{0}$ mode information was extracted from the impact AE to diagnose whether there was damage. The impact non-damage test layout is displayed in Figure 5b. The tested epoxy fibreglass plate had a thickness of 2 mm; the impact point was 20 cm away from PZT3 on the straight line between the three PZTs, which were spaced 4.7 cm apart. The sampling rate was 10^7^, with 10^4^ sampling points. An impact hammer was used in the first test (Figure 8). Due to the light weight of the hammer, no damage was visually observed at the impact point.

**Figure 9:**
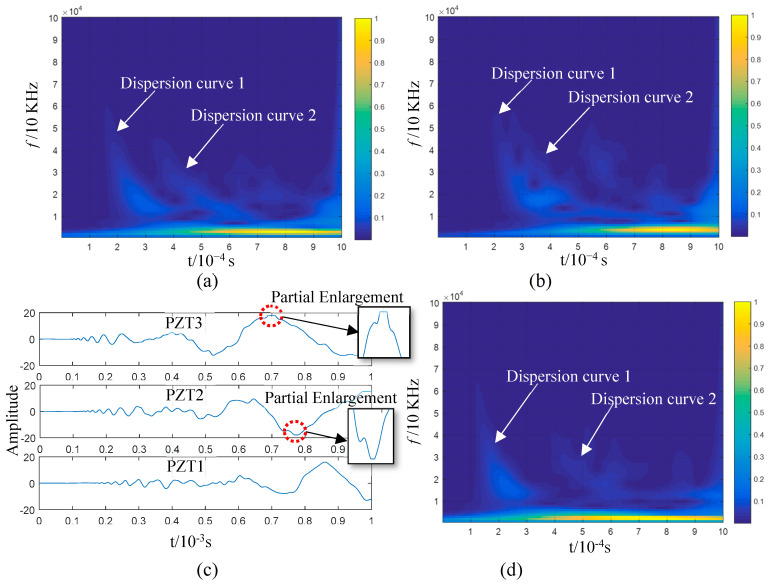
Acoustic emission (AE) and time–frequency analysis of an epoxy fibreglass plate after impact: (**a**) impact AE signal obtained using three piezoelectric ceramics; (**b**–**d**) wavelet time–frequency analysis of PZT3 (**b**), PZT2 (**c**), and PZT1 (**d**) signals.

**Figure 10:**
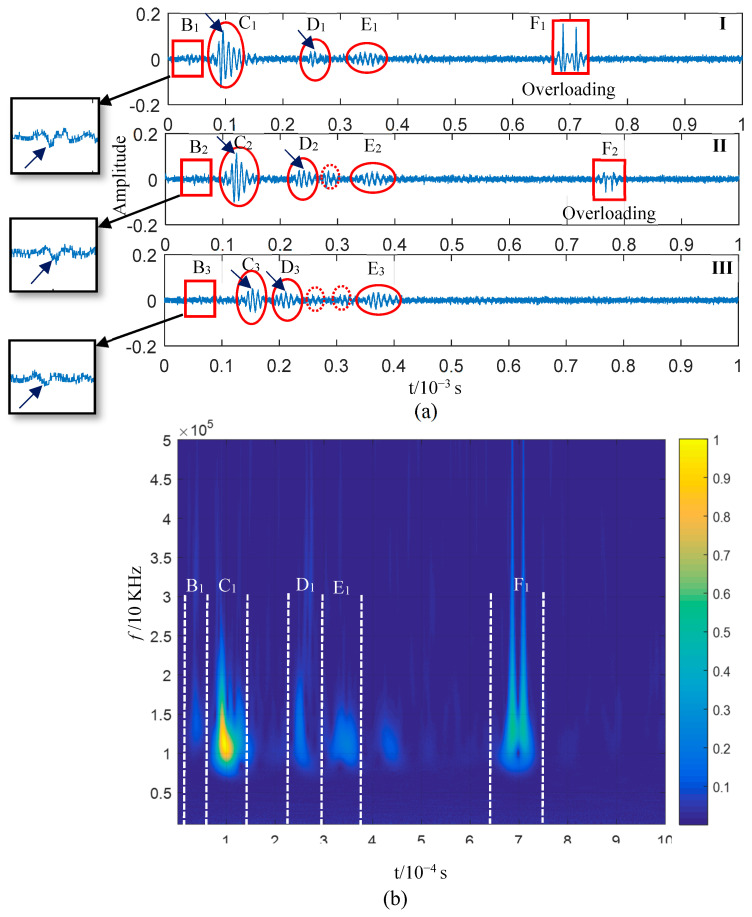
(**a**) High-pass zero-phase filtering of acoustic emission signals obtained using three piezoelectric ceramics, and (**b**) wavelet time–frequency analysis of sub-graph I shown in (**a**).

The resulting AE is shown in Figure 9a. No impact damage was observed on the plate structure through visual inspection, although the AE amplitude exceeded the voltage input range of the detection system (as shown in the insets of Figure 9a). The wavelet time–frequency analysis (Figure 9b,d) showed the dispersion characteristics of the wave in the signals collected by all the PZTs. However, the $S_{0}$-mode waveform could not be identified in the time domain and time–frequency plots. The high-frequency components of the AE’s $S_{0}$ mode were abundant according to the above analysis. Those in Figure 9a could be extracted to suppress the interference of low-frequency waves. On this basis, the mode information of the impact AEs could be analysed. The lower frequency limit of high-pass zero-phase filtering was set as 100 kHz. The filtering results are shown in Figure 10a, with the sub-graphs I–III presenting the filtering results of the three sensor signals.

If attenuation in the propagation process is considered, the AE was the component of the closest original signal obtained by PZT3. Therefore, the sub-graph I in Figure 10a was employed for a time–frequency analysis (Figure 10b). The wave packets in the F_1_ and F_2_ block diagrams in sub-graphs I and II are high-frequency components introduced via overload; they are not required for the analysis. The wave packets C_2_ and C_3_ represent the waveform when the wave packet C_1_ passes through PZT2 and PZT1. The wave packet velocity can be calculated if the extreme point (indicated by an arrow in the figure) is taken as the reference point (with the X-axis corresponding to 0.95 × 10^−3^ s, 1.236 × 10^−3^ s, and 1.532 × 10^−3^ s), as shown in Table 1. The frequency bands of the three wave packets (C_1_, C_2_, and C_3_) are within 100 and 150 kHz, according to the frequency-domain coordinates corresponding to the C_1_ wave packet in Figure 10b. Based on Figure 3b, the wave velocity range of the $A_{0}$ mode in this frequency band ranges from 1657 to 1830 m/s. The propagation velocities of C_1_, C_2_, and C_3_ calculated in Table 1 are consistent with this range, and the distribution of the three wave packets on the time axis is also consistent with dispersion curve 1 in Figure 9b–d. Therefore, the wave packets including C_1_, C_2_, and C_3_ probably correspond to the $A_{0}$ mode. In the test experiment illustrated in Figure 8, the type of impact was OP, and the AE generated by the impact was dominated by the $A_{0}$ mode and occurred for the first time. Hence, the first-mode wave packet in the high-pass filter signal (wave packets C_1_, C_2_, and C_3_ in Figure 10a) was generated by the impact.

**Figure 11:**
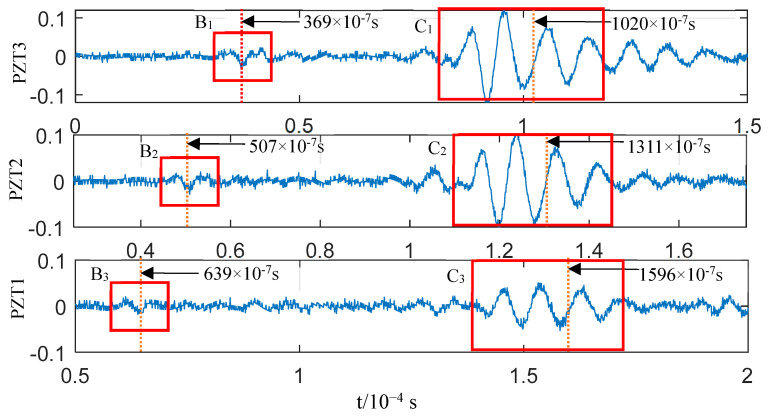
$A_{0}$-mode time point calculated according to the $S_{0}$-mode time point: the figure shows partially enlarged views of the first two wave packets (B_1_–B_3_, C_1_–C_3_) in sub-graphs I–III of Figure 10a.

The reason why the OP-generated AE is dominated by the $A_{0}$ mode was discussed in the previous section, and we do not intend to ignore the $S_{0}$ mode generated simultaneously because its energy is small. In the test illustrated in Figure 9, the component force parallel to the plate surface cannot be zero since a small amount of the $S_{0}$ mode wave packet exists in the impact AE. These two modes occur simultaneously when the plate is impacted according to the previous analysis. Since the $S_{0}$ mode propagation speed is greater than that of the $A_{0}$ mode, the two mode waves start to separate when they are generated, and the $S_{0}$ mode wave packet is always ahead of the $A_{0}$ mode wave packet on the time axis. A trace of the $S_{0}$ mode wave packet can be found by carefully analysing the waveforms before C_1_, C_2_, and C_3_. The waveforms marked in Blocks B_1_–B3 and their enlarged views are displayed in Figure 10a, showing their similarity. If the midpoint of these waves (the point indicated by an arrow in the insets) is taken as a reference point, the corresponding propagation velocity of the wave packet is ~3400 m/s (Table 1). The speed of the $S_{0}$ mode in Figure 3b is 3250 m/s. That is, the actual value matches the theoretical ones if the influences of noise, waveform attenuation, and propagation path on velocity are considered. Therefore, it has been confirmed that the above three waveforms correspond to the $S_{0}$ mode according to the propagation velocity of the B_n_ (n = 1,2,3) wave packets. However, this is not enough proof to demonstrate that the B_1_–B_3_ wave packets resulted from the impact; it must be proven that the B_1_–B_3_ and C_1_–C_3_ wave packets originated from the impact. The $A_{0}$-mode wave packets generated simultaneously were calculated according to the reference point time of the $S_{0}$-mode wave packets B_1_–B_3_ when the propagation distance and the velocity of the two modes were known. If the coordinates of these reference points fall within the C_1_–C_3_ wave packet period, the B_1_–B_3_ and C_1_–C_3_ wave packets will likely be generated from the impact. The actual propagation velocity between PZT3 and PZT1 (as shown in Table 1) can be obtained as the velocity of the $S_{0}$ and $A_{0}$ modes. The propagation distance is the distance from the impact position to the sensor (Figure 8). The time difference corresponding to the wave packet reference points of the impact-generated $A_{0}$ mode can be determined by calculating the TOA differences of the two mode wave packet reference points by referring to the point marked in the B_1_–B_3_ wave packets in Figure 10a. The results are summarised in Table 2. The time coordination of the $A_{0}$-mode reference point in Table 2 was compared with the time domain of the corresponding wave packets C_1_–C_3_ (Figure 11). The reference time of the $S_{0}$-mode wave packets B_1_–B_3_ and the corresponding time point of the calculated $A_{0}$-mode wave packets are indicated in Figure 11; the time area of the $A_{0}$-mode wave packets calculated using the $S_{0}$-mode ones is highly coincident with the C_1_–C_3_ wave packets. Therefore, the $S_{0}$-mode wave packets B_1_–B_3_ and the $A_{0}$-mode wave packets C_1_–C_3_ in Figure 10a likely originated simultaneously from the impact. The $S_{0}$- and $A_{0}$-mode wave packets in the AEs generated by the impact of external objects, which were separated at the moment of generation due to their different propagation velocities, were verified via the analysis of the experimental results. The $S_{0}$-mode wave packets will not be superimposed with the $A_{0}$ mode ones in the AE generated via the impact of external objects since their propagation velocity is greater than that of the $A_{0}$-mode wave packets.

Besides the typical Class II wave packets B_1_–B_3_ and C_1_–C_3_, Figure 10a also shows D_1_–D_3_ and E_1_–E_3_ wave packets with obvious amplitudes as well as other low-amplitude clutter (marked by dotted lines in sub-graphs Ⅱ and Ⅲ). D_1_–D_3_ are considered to be the reflected waves of C_1_–C_3_ from the time sequence distribution and velocity. Compared with D_2_ and D_3_, D_1_ has an abnormal waveform change, which was likely caused by clutter interference. The propagation direction of E_1_–E_3_ should be from PZT3 to PZT1 based on the time series layout. The propagation velocity of E_n_ measured using the reference point is between the $S_{0}$ and $A_{0}$ modes. A disturbance could not be proven in this paper since the wave packet is late in its time sequence, with abnormal amplitude changes, and no further discussion of it will be provided. No obvious $S_{0}$-mode wave packets were observed when considering the impact AE wave packets; in particular, no $S_{0}$-mode wave packets were discovered after the C_1_–C_3_ ones. The $A_{0}$-mode wave packets in the impact AE result from the impact of external objects. The analysis results in Figure 10a indicate that this impact did not damage the plate structure according to the mode characteristics of damage and external impact AE described in the previous section; this is consistent with the impact test conditions.

**Figure 12:**
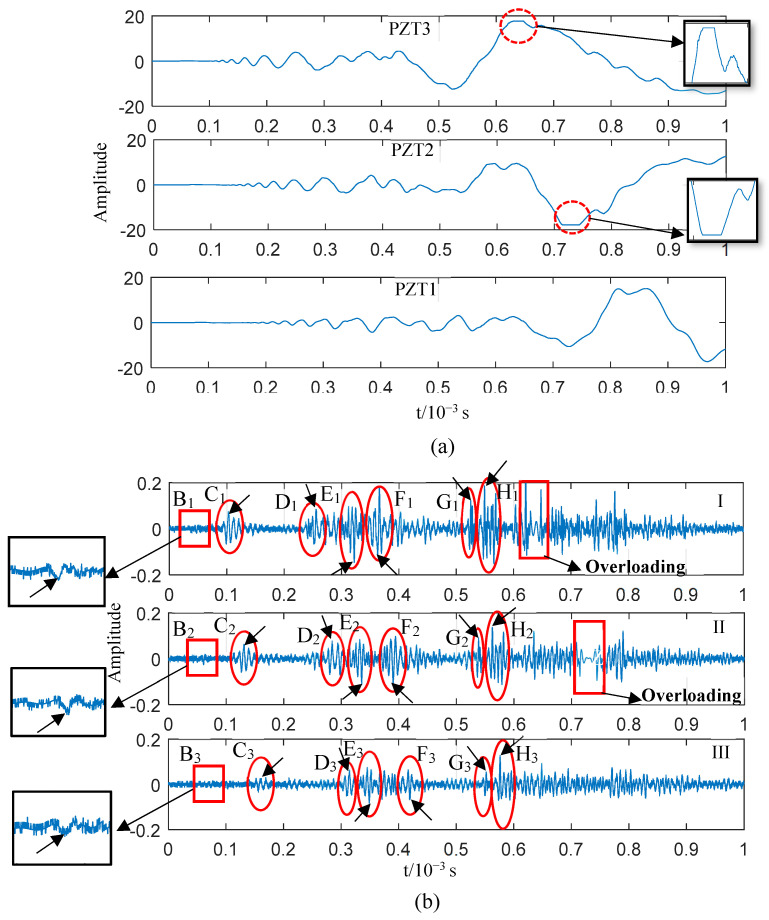
(**a**) Acoustic emission signals generated by pendulum impact and detected using the three piezoelectric ceramic devices. (**b**) The corresponding high-pass zero-phase filtering results.

When the impact tool was replaced by a pendulum mass for analysing the impact at the same position, damage could be observed on the epoxy fibreglass plate, as shown in Figure 8. The corresponding signals collected by the three PZTs are illustrated in Figure 12; these results are similar to those in Figure 9a in terms of the waveform. The AE generated by the impact of external matter was dominant, and the signal was overloaded with the waveform shown in dispersion. The difference is that the damage caused by the pendulum impact was visible. The high-frequency components of the three sensor signals (Figure 12a) were extracted using a zero-phase high-pass filter with a lower cut-off frequency of 100 kHz to further analyse the health information carried by the AEs generated by the pendulum impact; the results are shown in Figure 12b.

Compared with Figure 10a, the high-frequency signal obtained via filtering shown in Figure 12b has more wave packets. The sequence of wave packets in the three PZT signals is denoted as B_n_–H_n_. To confirm the modes of these wave packets, their propagation speeds were calculated. The typical points of each series of wave packets were selected (and marked with arrows in Figure 12b) as reference points for calculating the wave velocity, as shown in Table 3. According to the results, the B_n_ wave packets were in the $S_{0}$ mode, while the C_n_ ones were in the $A_{0}$ mode. The acoustic source of the B_n_ and C_n_ wave packets was the pendulum impact rather than the damage. This was explained in detail in the last impact test data analysis. In general, the high-frequency wave packets of these two modes could reach the sensor since the AEs were generated under the impact of external objects on the plate surface the first time. The wave velocity calculated for the D_n_ wave packets based on the reference points is consistent with the $A_{0}$ mode; their propagation direction is along the line drawn from PZT3 to PZT1 according to the time corresponding to the D_1_, D_2_, and D_3_ wave packets, excluding the reflection of B_n_ wave packets from this propagation direction. The amplitude of the D_n_ wave packets is not lower than that of the B_n_ ones. In this case, the D_n_ wave packets reflecting as B_n_ ones can also be excluded. Therefore, their source is different from that of the B_n_ wave packets, which can only be IP. It can be speculated, then, that the acoustic source of the D_n_ wave packets is delamination since there is no obvious $S_{0}$ mode before them.

Table 3 shows that the wave velocity of the E_n_ wave packets was approaching 3500 m/s, which is close to that of the $S_{0}$ mode; this indicates that the E_n_ wave packets were in the $S_{0}$ mode. The E_n_ wave packets were generated by the force applied parallel to the plane according to the relationship between the force direction and Lamb wave mode described before. The pendulum impacted the plate in a nearly perpendicular direction under the test conditions. The amplitude varies significantly between the E_n_ wave packets with the impacted $S_{0}$-mode wave (B_n_ wave packets). This means that the E_n_ component from the plate direction of the pendulum impact can be nearly excluded; hence, the E_n_ wave packets come from impact damage, either matrix or fibre breakage. For the remaining F_n_, G_n_, and H_n_ wave packets, according to the wave velocity in Table 3, it can be confirmed that the F_n_ ones belong to the $A_{0}$ mode and the others belong to the $S_{0}$ mode. The $A_{0}$-mode F_n_ wave packets previously had $S_{0}$-mode wave packets, which could only occur due to some kind of plate damage rather than generation via pendulum impact from the perspective of time sequence and amplitude. The $S_{0}$-mode G_n_ and H_n_ wave packets could merely have occurred due to damage rather than the pendulum impact. The typical wave packet (B_n_–H_n_) mode in the sequence diagram in Figure 12 has been confirmed. In addition, other wave packets may stem from damage. However, the acoustic source might be interfered with by the reflected waves at the edge since it is not situated along a straight line with respect to the three sensors; thus, it could not be recognised in the three sensor signals. Hence, it is not advantageous to further discuss the above modes.

The high-frequency components of the AEs reveal the acoustic source activity in the plate more clearly based on a comparison of the data before and after the impact AE filtering, as shown in Figure 9, Figure 10 and Figure 12. The impact AEs are dominated by the $A_{0}$ mode according to the impact wave test data shown in Figure 10a. The number of wave packets of the two modes is limited, with an orderly distribution at high frequencies (>100 kHz). The plate structure damage caused by the impact is a continuous process with multiple events and various damage types as per the distribution of the high-frequency wave packets in Figure 12b. Regarding the time sequence, damage-induced AEs occur significantly later than impact AEs. The high-frequency components, the wave packet mode ($S_{0}$ or $A_{0}$), and the distribution of the AE wave packet time sequence can be obtained by analysing the data from two tests for the multi-angle interpretation and accurate identification of damage-induced wave packets. The feasibility of the monitoring method for diagnosing the damage of thin plate structures has been proven since damage-induced AEs can be directly extracted from impact AEs.

## 5. Conclusions

AEs are essential for monitoring the health of plate structures. This paper proposed the direct extraction of damage-induced AEs from AE impacts in the damage diagnosis of thin plate structures because the monitoring method based on AE characteristics is susceptible to non-damage factors. Since there is no difference between impact and damage-induced AEs in nature, this paper recognised the two AEs by interpreting multiple factors, such as the mode ($S_{0}$ or $A_{0}$), amplitude, and time sequence distribution of the wave packets with the help of high-frequency-band zero-phase filtering based on the relationship between Lamb mode and force direction. This research consisted of the following four aspects.

1. The AE Lamb wave modes in thin plate structures were studied. The AE frequency in the plate was distributed below several hundred kHz, and the AE Lamb waves in the thin plate were within the mode cut-off range, with only two lowest-order modes ($S_{0}$ and $A_{0}$). The propagation velocity of the $S_{0}$ mode was almost unchanged within the mode cut-off range, while that of the $A_{0}$ mode showed significant dispersion.

2. The propagation velocities and modes of the stress waves generated by forces perpendicular and parallel to the plate surface were analysed using the mechanical equations of plate structures. The analysis showed that the stress wave generated by the force perpendicular to the plate surface corresponded to the $S_{0}$ mode, while that generated by the parallel force corresponded to the $A_{0}$ mode. The results of lead-breaking tests conducted on aluminium and epoxy fibreglass plates support this conclusion.

3. The difference between impact- and damage-induced AE in plate structures was analysed. First, the two AEs are separated in the time sequence. The plate structure undergoes deformation after absorbing kinetic energy until finally fracturing. Impact AEs arose before damage-induced AEs in the collected data. Second, the two AE modes have different characteristics. The smooth surface of the plate structure bears the impact of external objects in its vertical direction. The process of impact-induced damage is uncontrollable and characterised by multiple damage types, with forces acting in the parallel and perpendicular directions of the plate surface. As a result, impact AEs are dominated by the $A_{0}$ mode, whereas both the $S_{0}$ and $A_{0}$ modes are significant in damage-induced AEs.

4. This paper proposed considering the $S_{0}$ mode in impact AEs as an indication of whether the impact causes damage to a thin plate structure. This is because the $S_{0}$ mode in OP-generated AEs only occurs before the $A_{0}$ mode. Damage-induced AEs take place later than impact AEs. Thus, the generated $S_{0}$- and $A_{0}$-mode waves can only be superimposed with the $A_{0}$ mode low-frequency waves of the impact AEs. Therefore, if the $S_{0}$ mode wave packets extracted in the AE occur later than the OP-generated $A_{0}$-mode ones, the AE only occurs when the plate structure is damaged, and it is not an isolated event. The above conclusions were verified by analysing the impact test data of an epoxy fibreglass plate.

This study was performed in the cut-off range of the Lamb wave mode, which is applicable for thin (<2 mm) plate structures. If the frequency–thickness product exceeds the mode cut-off range, further discussions on the impact damage diagnosis method are recommended. The AE Lamb wave mode confirmed by the wave velocity was leveraged. The epoxy fibreglass plate used in the test is not significantly anisotropic; that is, it can be considered approximately isotropic for data analysis. If the fibre-laying angle of the composite plate is highly associated with the wave velocity, a detailed Lamb wave dispersion curve is required for discussing the AE mode.

## Figures and Tables

**Table 1:** Calculation of the propagation speed of wave packets B_n_ and C_n_ (n = 1, 2, 3).

Wave Packet	Lower Label (10^−7^ s)			Speed between Two Sensors (m/s)		Mode
	1	2	3	PZT3 and PZT2	PZT2 and PZT1	
B	369	507	639	3406	3561	$S_{0}$
C	956	1236	1532	1680	1590	$A_{0}$

**Table 2:** Reference point time of $S_{0}$ mode calculated based on its reference point.

		Distance between Impact Position and Sensor (m)	Figure 3b Wave Velocity (m/s)		$\mathrm{Reference}\;\mathrm{Point}\;\mathrm{of}\;S_{0}$ Mode (10^−7^ s)	Calculation Results	
Sensors							
						Time Difference of Arrival Sensor in Two Modes (10^−7^ s)	$\mathrm{Reference}\;\mathrm{Point}\;\mathrm{of}\;A_{0}$ Mode (10^−7^ s)
			$S_{0}$	$A_{0}$			
PZT3		0.200	3482	1632	369	651	1020
PZT2		0.247			507	804	1311
PZT1		0.294			639	957	1596

**Table 3:** Wave packet velocity calculation, pattern recognition, and determination of acoustic sources in pendulum impact.

Wave Packet	Reference Point Coordinates of the Wave Packet (10^−7^ s)			Wave Velocity between PZT3 and PZT2 (m/s)	Wave Velocity between PZT2 and PZT1 (m/s)	Mode	Sound Source
	Subscript 1	Subscript 2	Subscript 3				
B	477	611	741	3507	3615	$S_{0}$	Impact
C	1026	1306	1605	1679	1572	$A_{0}$	Impact
D	2558	2842	3144	1655	1556	$A_{0}$	Damage
E	3221	3355	3490	3508	3481	$S_{0}$	Damage
F	3634	3902	4194	1754	1610	$A_{0}$	Damage
G	1610	5385	5522	3561	3431	$S_{0}$	Damage
H	5492	5623	5762	3588	3381	$S_{0}$	Damage

## Data Availability

Not applicable.
